# Bioinspired Heterocyclic Partnership in a Cyanine-Type Acidichromic Chromophore

**DOI:** 10.3390/molecules25173817

**Published:** 2020-08-21

**Authors:** Maria Laura Alfieri, Lucia Panzella, Marco d’Ischia, Alessandra Napolitano

**Affiliations:** Department of Chemical Sciences, University of Naples “Federico II”, I-80126 Naples, Italy; marialaura.alfieri@unina.it (M.L.A.); panzella@unina.it (L.P.); dischia@unina.it (M.d.)

**Keywords:** benzothiazine-based chromophores, heterocyclic aromatic aldehyde, cyanine dyes, acidichromism, pH sensors

## Abstract

A new red hair-inspired 1,4-benzothiazine-based scaffold is disclosed herein, built upon a modular D-π-A architecture via condensation of the easily accessible 3-phenyl-2*H*-1,4-benzothiazine with indole-3-carboxaldehyde. The compound was obtained in around 50% yields and was characterized by complete spectroscopic analysis. The new benzothiazine-based cyanine displayed a characteristic reversible acidichromic behavior with a marked bathochromic shift upon acidification. The chromophore resisted at least fifteen hydrochloric acid/sodium hydroxide cycles without appreciable alterations. The expedient and scalable synthetic procedure together with the pH sensitive chromophoric properties would make the new compound an attractive prototype for novel modular chromophore for pH-sensing and other applications.

## 1. Introduction

The design and build-up of organic-based chromophores exhibiting tailored electronic and optical features has become an active research field over the past decade. Among different applications, particular interest has been focused on optoelectronics (e.g., dye sensitized solar cells, photo- and chemical-switching systems, organic light emitting devices) [1,2,3,4,5], organic electronics, such as organic semiconductors [6,7,8,9], imaging technologies (e.g., electrophotography, thermal printing, and ink-jet printing) [10], and biomedicine (e.g., fluorescent sensors and anticancer treatments or photodynamic therapy) [11,12,13,14,15,16,17].

In addition to chemical stability and intense absorption in the visible region, desirable properties of functional dyes include any change of the chromophoric properties, such as photochromism or solvatochromism, that is associated with a modification of the external parameters or aggregation state [18,19,20,21,22]. 

An interesting nature-inspired class of compounds featuring most of the requisites for technological applications are cyanine dyes, which are structurally related to the betacyanin pigments occurring in fruits and vegetables and exhibiting red purple chromophores. These dyes feature an imine and an enamine type site arranged to form a push-pull system. Because of their excellent photophysical properties, such as high molar extinction coefficients and high fluorescence quantum yields, outstanding biocompatibility, and low toxicity to living systems, cyanines are good candidates for developing chemosensors [23,24,25,26].

**Figure 1:**
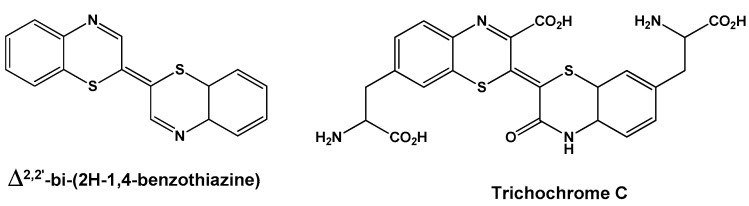
Representative structures of trichochromes.

Recently, novel cyanine-type dyes, designated trichocyanines [27], have been built upon a modular D-π-A architecture which incorporates ring systems inspired by the Δ^2,2′^-bi-(2H-1,4-benzothiazine) structural core of trichochromes: unique pH-dependent aminoacidic pigments occurring in red human hair and avian feathers [28,29,30] (Figure 1). The most characteristic feature of trichochromes is their doubly cross-conjugated yellow-to-red chromophore, which undergoes a marked bathochromic shift to an intense blue coloration in acid as a result of the protonation of the benzothiazine nitrogen that enhances its acceptor character [31,32,33]. 

**Figure 2:**
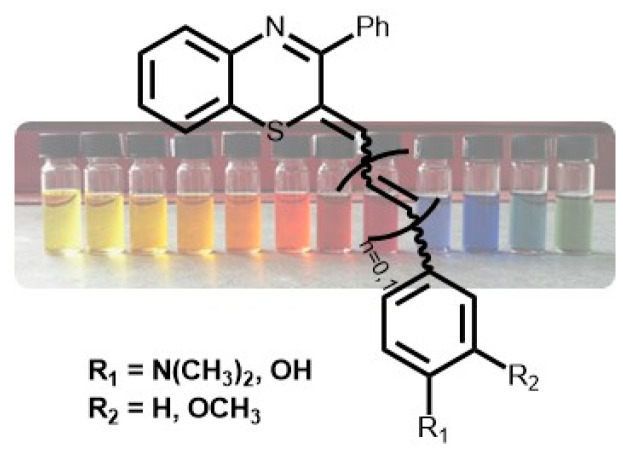
Structure of the trichocyanines synthesized and color variation associated to pH changes in the range 1–12 [27].

The trichocyanines, in particular, showed an extension of the conjugated system compared to that exhibited by Δ^2,2’^-bi-(2*H*-1,4-benzothiazine) [32,34], which expectedly resulted in a larger bathochromic shift in acid. Therefore, variation of the π-bridge could be recognized as a facile and effective approach for developing push-pull chromophores with specific properties and easy tunability by modulating the electron-donating substituent(s) on the phenyl ring (Figure 2). 

Starting from this background it was argued that novel functional chromophores could be obtained by reaction of the nucleophilic enamine-type C2 position of the 2*H*-1,4-benzothiazine moiety [35,36,37] with carboxaldehydes of nitrogenous heterocyclic aromatic systems. This would allow the build-up of a variant of the benzothiazine-based cyanine chromophore, with the benzothiazine nitrogen acting as acceptor and the nitrogen of the heterocyclic aromatic system as donor component. 

In this work this rationale was tested through the preparation and characterization of a new benzothiazine-based system by reaction of the easily accessible 3-phenyl-2*H*-1,4-benzothiazine [35] with indole-3-carboxaldehyde. The ease of the synthetic procedure and the peculiar acidichromic behavior open new perspectives for the design and exploitation of nature-inspired chromophores for technological applications.

## 2. Results and Discussion

### 2.1. Synthesis and Structural Characterization of Cyanine ***1***

**Scheme 1:**
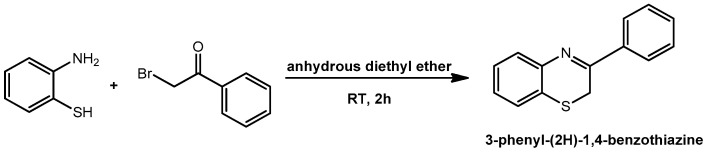
Synthesis of 3-phenyl-(2*H*)-1,4-benzothiazine.

First, the 3-phenyl-(2*H*)-1,4-benzothiazine was synthesized using a previously developed procedure [35] in which the *o*-aminothiophenol was reacted with phenacyl bromide in anhydrous diethyl ether. In 2 h a complete conversion of the starting materials was obtained and the compound separated from the reaction mixture as a yellow solid in pure form (ca. 80% yields) (Scheme 1). 

In the first series of experiments, the reaction between 3-phenyl-2*H*-1,4-benzothiazine and indole-3-carboxaldehyde was optimized starting from the conditions previously developed for the reaction with benzaldehyde or cinnamaldehyde in acetonitrile in the presence of HCl [27] (Table 1). TLC analysis of the reaction mixtures using different amounts of the acid showed that concentrations as high as 2.5 M were required to achieve a satisfactory and rapid consumption of the benzothiazine and to minimize by-product formation. In addition, the reaction proved faster, with an almost complete consumption of the starting reagent at 3 h, at which point the temperature was raised from 25 °C to 70 °C. Notably, this reaction time was significantly shorter than that used in previously reported procedures [27]. The stoichiometric ratio of the two reagents was varied in the range benzothiazine/aldehyde from 0.5:1 to 1:2. A 1:1.5 molar ratio proved to be an optimal condition to favor product formation over side reactions such as self-coupling of the benzothiazine.

**Scheme 2:**
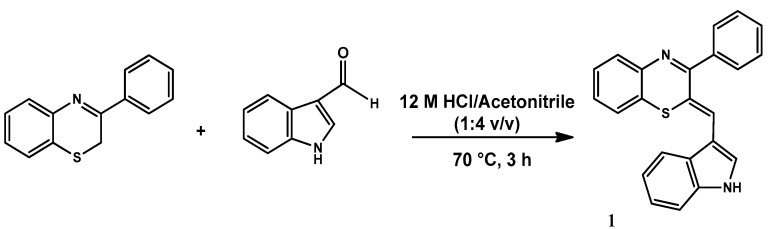
Synthesis of 2-((1*H*-indol-3-yl)methylene)-3-phenyl-2*H*-1,4-benzothiazine) (**1**).

On this basis, for synthetic purposes, the reaction was run on a preparative scale in 12 M HCl/acetonitrile 1:4 *v*/*v*, at 70 °C, starting from hundreds milligrams of 3-phenyl-(2*H*)-1,4-benzothiazine. After 3 h TLC analysis showed the presence of a main component (*R*_f_ = 0.42, eluant cyclohexane/ethyl acetate 8:2 *v*/*v*) together with minor components. After removal of acetonitrile under reduced pressure, the aqueous acid mixture was extracted with ethyl acetate. The main product of the reaction, yellow in color, was isolated in pure form by silica gel column chromatography fractionation in around 50% yield and subjected to a complete spectroscopic characterization that led to its formulation as 2-((1*H*-indol-3-yl)methylene)-3-phenyl-2*H*-1,4-benzothiazine) (**1**) shown in Scheme 2.

MALDI mass spectrometry analysis in positive reflectron mode (Figure S1) indicated a pseudomolecular ion peak [M + H]^+^ at *m*/*z* 353, as expected for the condensation product **1**. Complete spectral characterization (Figures S2–S6) confirmed structure **1** proposed for the compound. The ^1^H NMR spectrum (Figure S2) showed a well-defined pattern of resonances attributable to the 1,4-benzothiazine and indole moieties, the phenyl substituent, and the diagnostic singlet for the exocyclic double bond at 7.30 ppm. In addition, the NOESY spectrum (Figure S7) allowed the assignment of the Z configuration to the double bond formed in the condensation reaction, showing a cross-peak between the singlet at 7.30 ppm and the multiplet at 7.82 ppm due to the protons of the phenyl ring ortho to the substitution position. A 2D NMR analysis allowed complete assignment of proton and carbon resonances (Figure S8). 

### 2.2. Characterization of the pH-Dependent Chromophore

**Figure 3:**
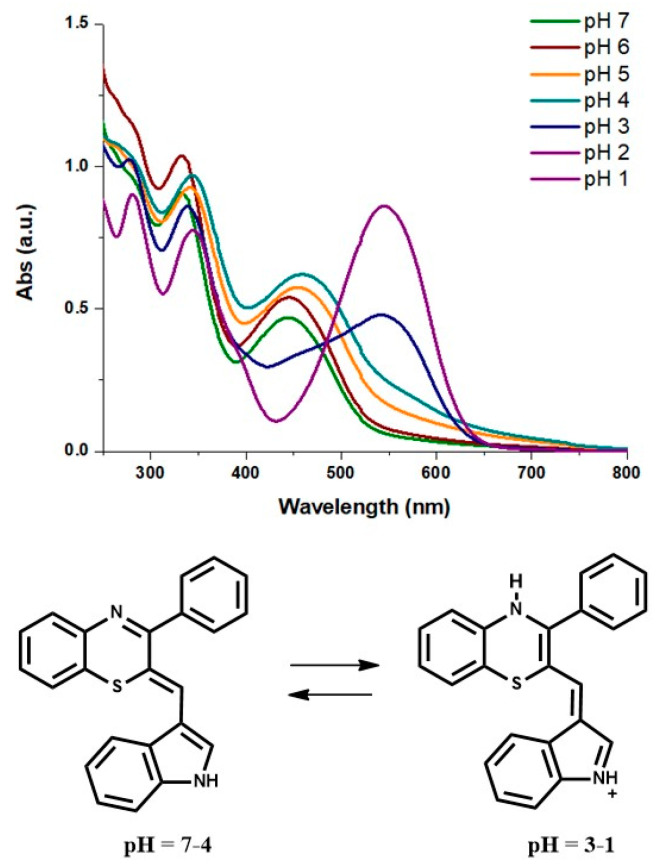
**Top**: UV-vis absorption spectra of cyanine **1** dissolved in methanol and diluted to 0.1 mM in 50 mM sodium phosphate buffer at different pHs. **Bottom**: protonation equilibrium of cyanine **1**.

The chromophoric properties of cyanine **1** were systematically investigated over the whole pH range. The cyanine proved freely soluble in organic solvents like chloroform or alcohols and also exhibited a good solubility in ethanol or methanol/water 70:30 and 50:50 mixtures. For the purpose of spectrophotometric experiments, a mother solution in methanol at 5 mM was prepared and then properly diluted in the buffer solutions. At neutral pHs, absorption maxima centered at 332 and 444 nm were observed. At acidic pH below 3 the band at higher wavelength underwent a large bathochromic shift (544 nm) and a marked hyperchromic effect (Figure 3). This shift is likely due to the protonation of the imine nitrogen of the benzothiazine moiety whose relatively high basicity stems from the stabilization of the resulting iminium ion provided by the indole nitrogen (Figure 3). The molar extinction coefficients (ε) for the neutral (444 nm) and acid (544 nm) forms were found to be 5403 ± 48 M^−1^ cm^−1^ and 12461 ± 29 M^−1^ cm^−1^ respectively. 

**Figure 4:**
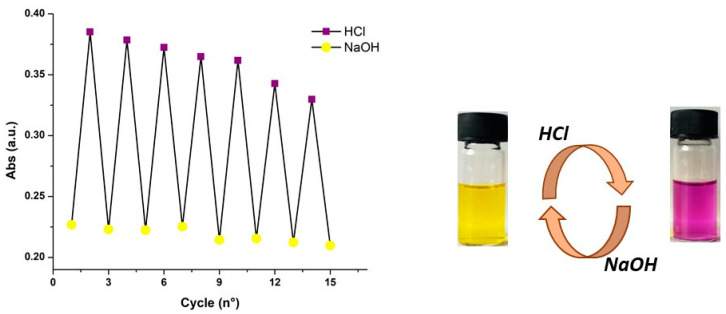
Changes of absorbance at 544 nm and digital pictures of **1** at 0.1 mM following repeated sequential addition of HCl and NaOH solution, up to pH 2.0 or 7.0 respectively.

Notably, the acid/base shift proved fully reversible over at least fifteen HCl/NaOH addition cycles (Figure 4). 

The chromophore of the cyanine **1** in the neutral form proved to be affected by solvent changes only to a limited extent with a maximum bathochromic shift of around 15 nm passing from chloroform to water (Figure S9).

In other experiments the fluorescence properties of cyanine **1** at different pHs were also evaluated. The product showed a moderate fluorescence at pH 7.0 by irradiation at 332 nm that was quenched in acid (pH = 2.0), without the generation of any other fluorophore (Figure S10). 

**Figure 5:**
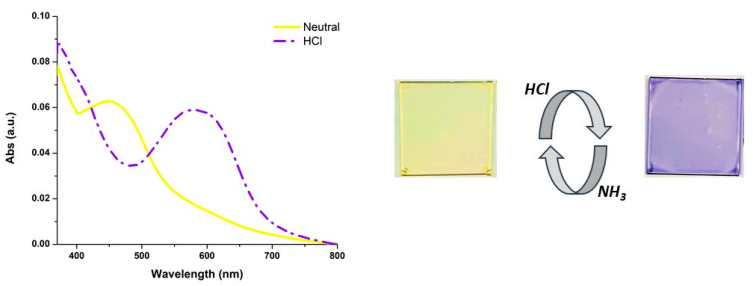
UV-vis spectra and digital pictures of glass slides coated with **1** (5 mM) before and after exposure to acidic vapors.

Cyanine **1** in methanol was easily deposited by spin coating onto different substrates (e.g., glass slides), yielding homogeneous thin films yellow in color which rapidly turned violet after exposure to gaseous hydrochloric acid (Figure 5). Notably, the shift proved to be completely reversible upon exposure to gaseous ammonia. Exposure to vapors of organic acids such as trifluoroacetic acid or acetic acid produced a similar though less marked color shift (Figure S11).

**Figure 6:**
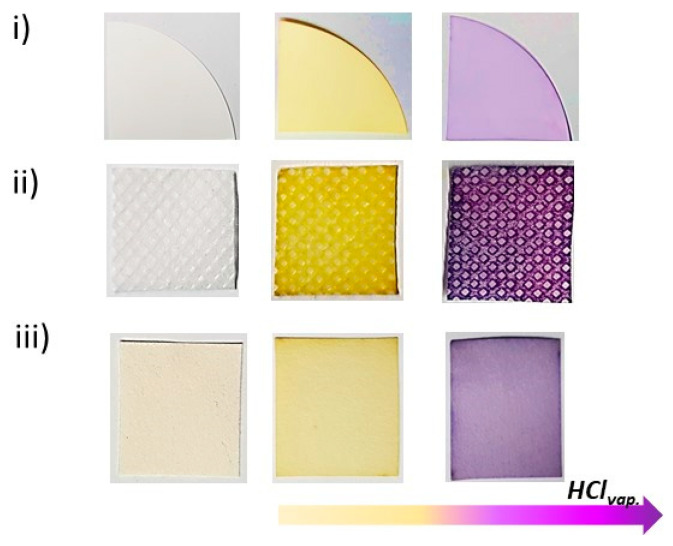
Nylon (**i**), cotton (**ii**), and filter paper (**iii**) dyed by dipping into a 5 mM methanol solution of cyanine **1**. Rows (i)–(iii): Left: before dyeing, middle: dyeing with **1**; right: after exposure to acidic vapors.

In further experiments the potential of cyanine **1** as dye was evaluated using materials much different in nature spanning from nylon to cotton and filter paper. As apparent from pictures in Figure 6 in all cases the materials were homogeneously dyed and underwent a rapid and well appreciable shift of the hue on exposure to acidic vapors. 

## 3. Materials and Methods

Phenacyl bromide, indole-3-carboxaldehyde, and 2,5-dihydroxybenzoic acid were purchased from Sigma-Aldrich; *o*-aminothiophenol was purchased from Fluka. All solvents were obtained from Carlo Erba. Analytical TLC were carried out on silica gel plates (0.25 mm) from Merck using cyclohexane/ethyl acetate 8:2 *v*/*v* as the eluent. 

UV-vis spectra were recorded with a Jasco V-560 UV-vis spectrophotometer.

Fluorescence spectra were recorded with a Jasco FP-750 spectrofluorometer.

^1^H NMR and ^13^C NMR spectra were recorded in CDCl_3_ at 400 MHz on a Bruker 400 MHz spectrometer. ^1^H,^1^H COSY, ^1^H,^13^C HSQC, and ^1^H,^13^C HMBC were run at 400 MHz using Bruker standard pulse programs.

Positive Reflectron MALDI spectra were recorded on an AB Sciex TOF/TOF 5800 instrument using 2,5-dihydroxybenzoic acid as the matrix. One milliliter of the analyte, dissolved in chloroform, was premixed with 1 mL of the matrix in a centrifuge tube, and then 2 μL of the resulting mixture was pipetted on the MALDI target plate and air-dried for MALDI-ToF MS analysis. The laser was operated at 3700 Hz and the spectrum represented the sum of 15,000 laser pulses from randomly chosen spots per sample position. Raw data were analyzed using the computer software provided by the manufacturers and were reported as monoisotopic masses.

Spin-coating experiments were conducted with a spin-coater Laurell WS-650MZ-23NPP/LITE. The spin coating parameters were set as the following: α = 500 rpm/s, ω = 2000 rpm, t = 30 s. Glass slides were cleaned by sonication in ethanol for 10 min, then washed with isopropanol and dried with a compressed air flow. 

### 3.1. Synthesis of 3-Phenyl-(2H)-1,4-benzothiazine

The 3-phenyl-(2*H*)-1,4-benzothiazine was prepared according to Santacroce et al. [35]. A solution of *o*-aminothiophenol (1.1 mL, 8.7 mmol) in anhydrous ethyl ether (5 mL) was treated at room temperature with a solution of phenacyl bromide (2.2 g, 11 mmol) in anhydrous ethyl ether (25 mL) under magnetic stirring for 2 h. The yellow solid that separated was filtered, washed with ethyl ether, and then dried under vacuum to give the pure product (82% yield). The purity was checked by ^1^H NMR analysis.

### 3.2. Synthesis of 2Z-((1H-Indol-3-yl)methylene)-3-phenyl-2H-1,4-benzothiazine)

3-phenyl-2*H*-1,4-benzothiazine (200 mg, 0.62 mmol) dissolved in acetonitrile/12 M hydrochloric acid (4:1 *v*/*v*) (16 mL) was treated with indole-3-carboxaldehyde (103 mg, 0.89 mmol) under stirring at 70 °C for 3 h. After 3 h, at complete consumption of the benzothiazine (TLC analysis), acetonitrile was removed under reduced pressure and the mixture was extracted with ethyl acetate (3 × 50 mL). The organic layers were dried over anhydrous sodium sulphate and taken to dryness. The residue thus obtained (150 mg) was dissolved in chloroform (2 mL) and fractionated by silica gel column chromatography (eluent cyclohexane/ethyl acetate 80:20 *v*/*v*) to give **1** in pure form (120 mg, 55% yield).

UV: λ_max_ (CH_3_OH) 332 nm, 444 nm; MALDI-MS: C_23_H_16_N_2_S *m*/*z* 353 ([M + H]^+^); ^1^H NMR (CDCl_3_) δ (ppm): 7.07 (m, 1H), 7.10 (m, 1H), 7.20 (m, 3H), 7.30 (s, 1H), 7.40 (m, 2H), 7.49 (d, 4H), 7.82 (m, 2H), 7.95 (s, 1H), 7.99 (s, 1H); ^13^C NMR (CDCl3) δ (ppm): 111.3 (CH), 111.4 (CH), 112.3 (C), 117.1 (C), 118.3 (CH), 120.4 (CH), 123.0 (C), 123.5 (C), 124.4 (CH), 126.0 (CH), 126.1 (CH), 126.7 (CH), 127.1 (C), 127.3 (CH), 128.4 (CH × 2), 129.3 (CH × 2), 129.4 (CH), 129.8 (CH), 135.3 (C), 140.4 (C), 162.6 (C).

### 3.3. Film Formation with ***1*** and Dyeing Experiments 

Cyanine **1** dissolved in methanol at 5 mM was deposited on glass slides using a spin-coater. For dyeing experiments, the proper fabric was dipped into a solution of **1** in methanol at 5 mM for 1 min and then allowed to dry in air. In all cases the dyed materials were exposed to acid vapors at room temperature (12 M HCl, trifluoroacetic acid, glacial acetic acid). 

## 4. Conclusions

In recent years cyanine dyes have largely been exploited as biological reporters and in other technological applications by virtue of their peculiar chromophoric and fluorescence properties. Inspired by Δ^2,2′^-bibenzothiazine photochromic and acidichromic chromophore of red human hair pigments trichochromes, a new class of cyanine dyes termed trichocyanines was designed in which a highly tunable cyanine-type chromophore was implemented using the benzothiazine nitrogen as the acceptor moiety at high basicity, allowing for a marked bathochromic shift even at slightly acidic pHs. Herein we have synthesized a new 1,4-benzothiazine based cyanine chromophore by condensation of indole-3-carboxaldehyde with 3-phenyl-1,4-benzothiazine at the nucleophilic 2 position. The cyanine thus obtained, characterized as 2*Z*-((1H-indol-3-yl)methylene)-3-phenyl-2H-1,4-benzothiazine) by complete spectral analysis, exhibited a reversible acidichromic behavior with a marked bathochromic shift upon acidification from yellow (444 nm at neutral pH) to violet (544 nm at pH 2) with molar extinction coefficient in the order of 12,000 M^−1^ cm^−1^ (acid form). The ease and scalability of the synthetic procedures and the preliminary assessment of the dyeing ability of the cyanine would encourage exploitation of the new acidichromic system as a core unit for pH sensing devices and related applications. The results reported herein are also of interest for the possibility of developing adhesive formulations of the reported cyanine involving, e.g., mussel-inspired polydopamine films and related coatings, in order to assess their possible exploitation for pH sensing on various surfaces underwater.

## Figures and Tables

**Table 1:** Optimization of reaction condition for the synthesis of cyanine **1**.

Entry	Benzothiazine/Aldehyde (Molar Ratio)	Solvent	Temperature (°C)	Time (h)
**1**	**1:1**	**CH_3_CN/0.5 M HCl**	**r.t.**	**24**
**2**	**1:1**	**CH_3_CN/1.5 M HCl**	**r.t.**	**24**
**3**	**1:1**	**CH_3_CN/2.5 M HCl**	**r.t.**	**24**
**4**	**1:1**	**CH_3_CN/2.5 M HCl**	**45**	**8**
**5**	**1:1**	**CH_3_CN/2.5 M HCl**	**70**	**3**
**6**	**0.5:1**	**CH_3_CN/2.5 M HCl**	**70**	**3**
**7**	**1:2**	**CH_3_CN/2.5 M HCl**	**70**	**3**
**8**	**1:1.5**	**CH_3_CN/2.5 M HCl**	**70**	**3**

## Supplementary Materials

The following are available online, Figure S1: Segmental spectrum of MALDI-ToF (*m*/*z*: 250–800 Da) characterization of compound **1**. Figure S2: ^1^H NMR spectrum of **1** in CDCl_3_. Figure S3: ^13^C NMR spectrum of compound **1** in CDCl_3_. Figure S4: ^1^H,^1^H COSY spectrum of compound **1** (CDCl_3_). Figure S5: ^1^H,^13^C HSQC spectrum of compound **1** (CDCl_3_). Figure S6: ^1^H,^13^C HMBC spectrum of compound **1** (CDCl_3_). Figure S7: NOESY spectrum of compound **1** (CDCl_3_). Figure S8: ^1^H (black) and ^13^C (red) NMR resonances of cyanine **1**. Figure S9: UV-vis absorption spectra of cyanine **1** (100 µM) in different organic solvents. Figure S10: Emission spectra of the neutral and protonated forms of cyanine **1**. Figure S11: UV-vis spectra and digital pictures of glass slides, coated with **1**, after exposure to acidic vapors.
