# Supplementary material for: Bioinspired Heterocyclic Partnership in a Cyanine-Type Acidichromic Chromophore
Source: Molecules. 2020 Aug 21;25(17):3817. doi: 10.3390/molecules25173817 (PMC7504425; doi:10.3390/molecules25173817)
Supplement: Supplementary file 1 [file molecules-25-03817-s001.pdf]

Bioinspired Heterocyclic Partnership in a Cyanine-type Acidichromic Chromophore

Maria Laura Alfieri, Lucia Panzella, Marco d'Ischia and Alessandra Napolitano*

Department of Chemical Sciences, University of Naples "Federico II", Naples, Italy; marialaura.alfieri@unina.it (M.L.A.); panzella@unina.it (L.P.); dischia@unina.it (M.d.I.).

* Correspondence: alesnapo@unina.it

Table of contents

**Figure S1.** Segmental spectrum of MALDI-ToF (*m*/*z*: 250-800 Da) characterization of compound **1**	**S3**
**Figure S2.** ^{1}H NMR spectrum of **1** in $CDCl_3$	**S4**
**Figure S3.** ^{13}C NMR spectrum of compound **1** in $CDCl_3$	**S5**
**Figure S4.** $^{1}H,^{1}H$ COSY spectrum of compound **1** ($CDCl_3$)	**S6**
**Figure S5.** $^{1}H,^{13}C$ HSQC spectrum of compound **1** ($CDCl_3$)	**S7**
**Figure S6.** $^{1}H,^{13}C$ HMBC spectrum of compound **1** ($CDCl_3$)	**S8**
**Figure S7.** NOESY spectrum of compound **1** ($CDCl_3$).	**S9**
**Figure S8.** ^{1}H (black) and ^{13}C (red) NMR resonances of cyanine **1**	**S9**
**Figure S9.** UV-vis absorption spectra of cyanine **1** (100 μM) in different organic solvents	**S10**
**Figure S10.** Emission spectra of the neutral and protonated forms of cyanine **1**	**S11**
**Figure S11.** UV-vis spectra and digital pictures of glass slides, coated with **1**, after exposure to acidic vapours	**S12**

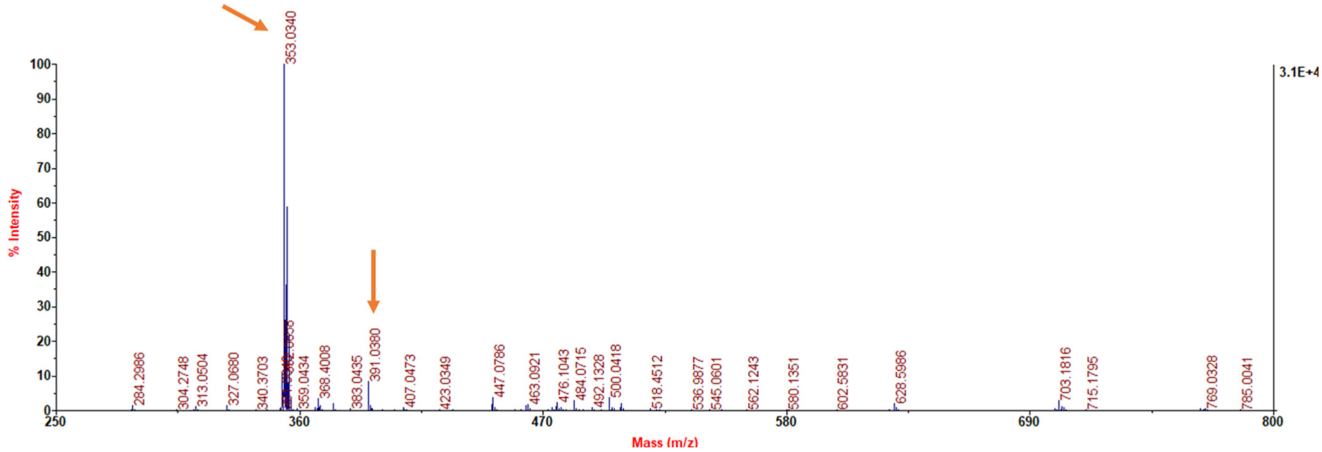

Figure S1. Segmental spectrum of MALDI-ToF (*m/z*: 250-800 Da) characterizations of compound **1**. Arrows indicate signals at $[M+H]^+$ = *m/z* 353 and $[M+K]^+$ = *m/z 391* due to the compound **1,** while other signals are due to matrix or impurities.

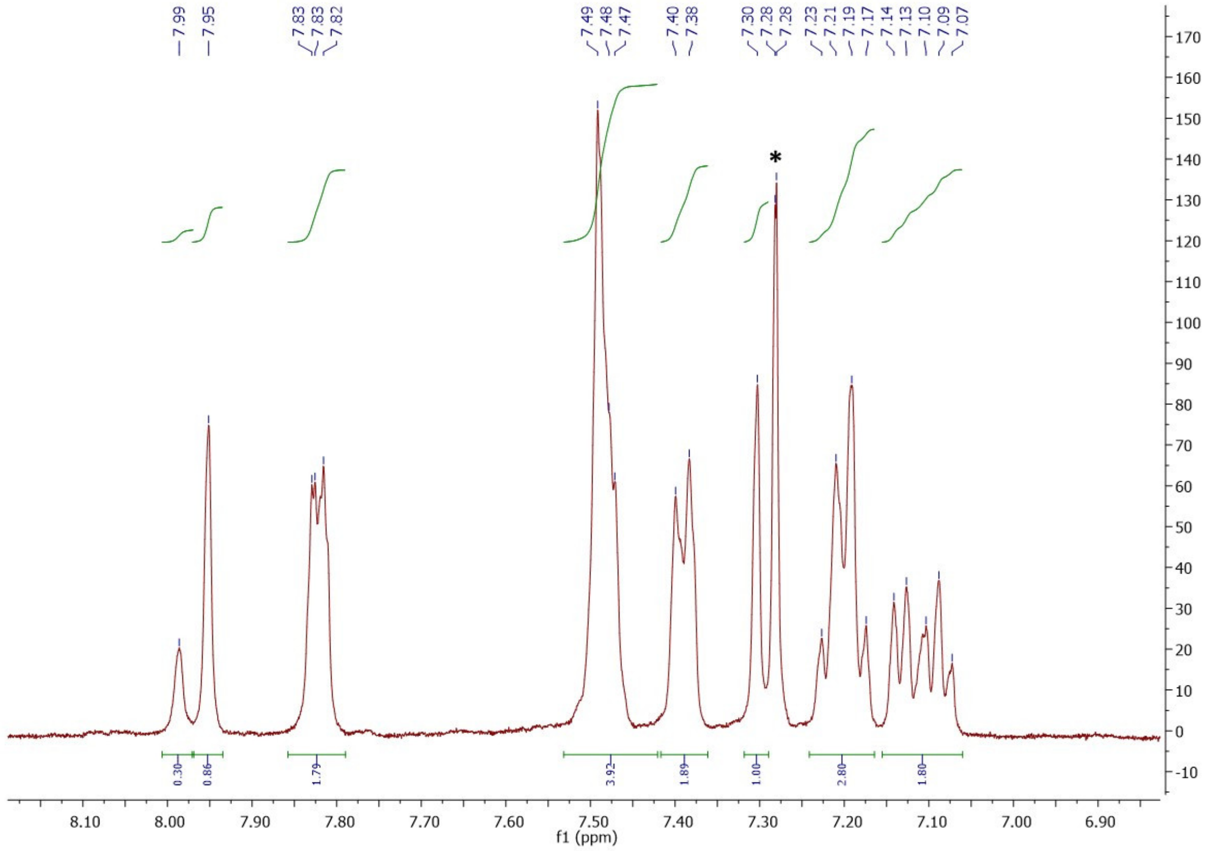

Figure S2. ^{1}H NMR spectrum of **1** in $CDCl_3$. Asterisk indicates the $CHCl_3$ proton resonance.

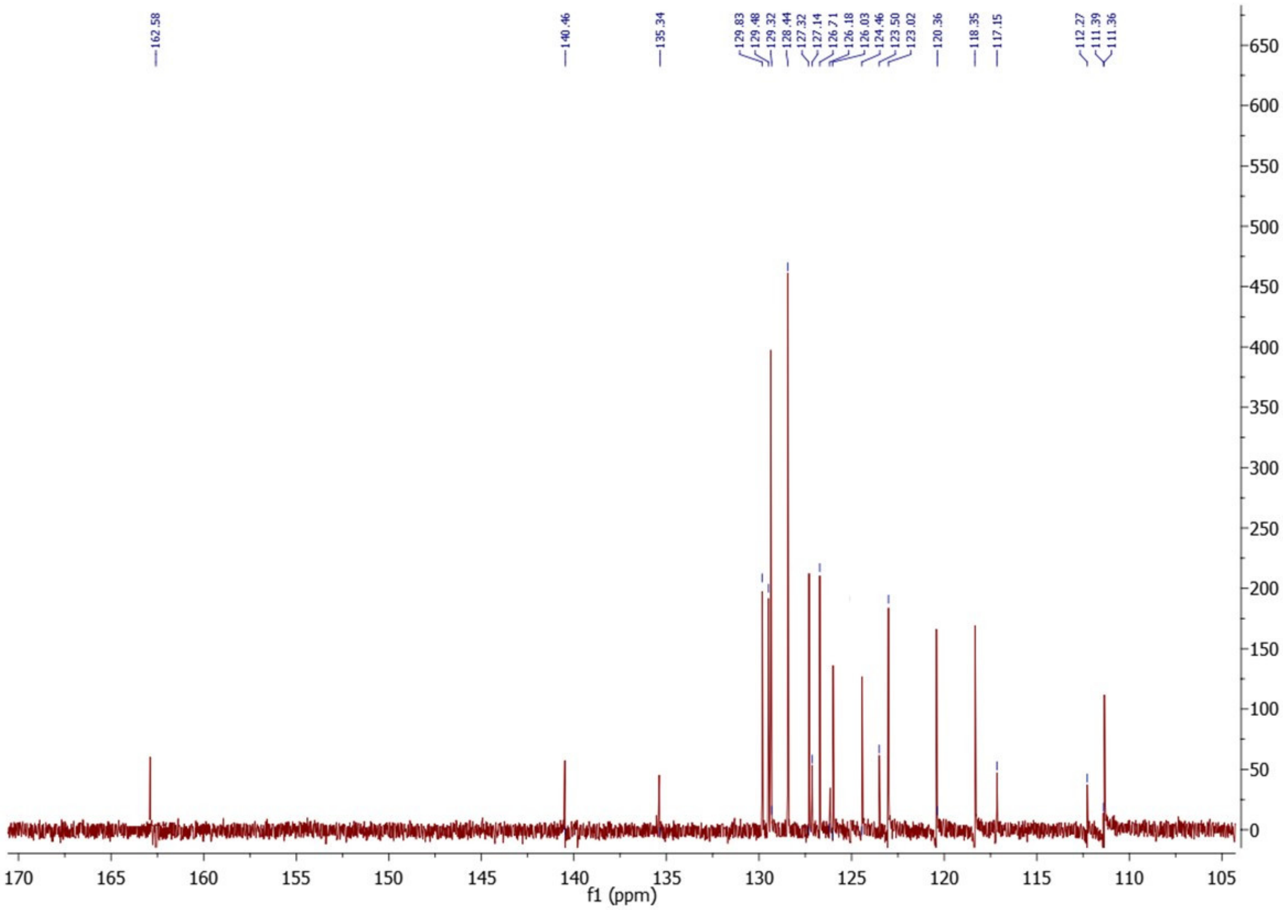

Figure S3. ^{13}C NMR spectrum of compound **1** in $CDCl_3$.

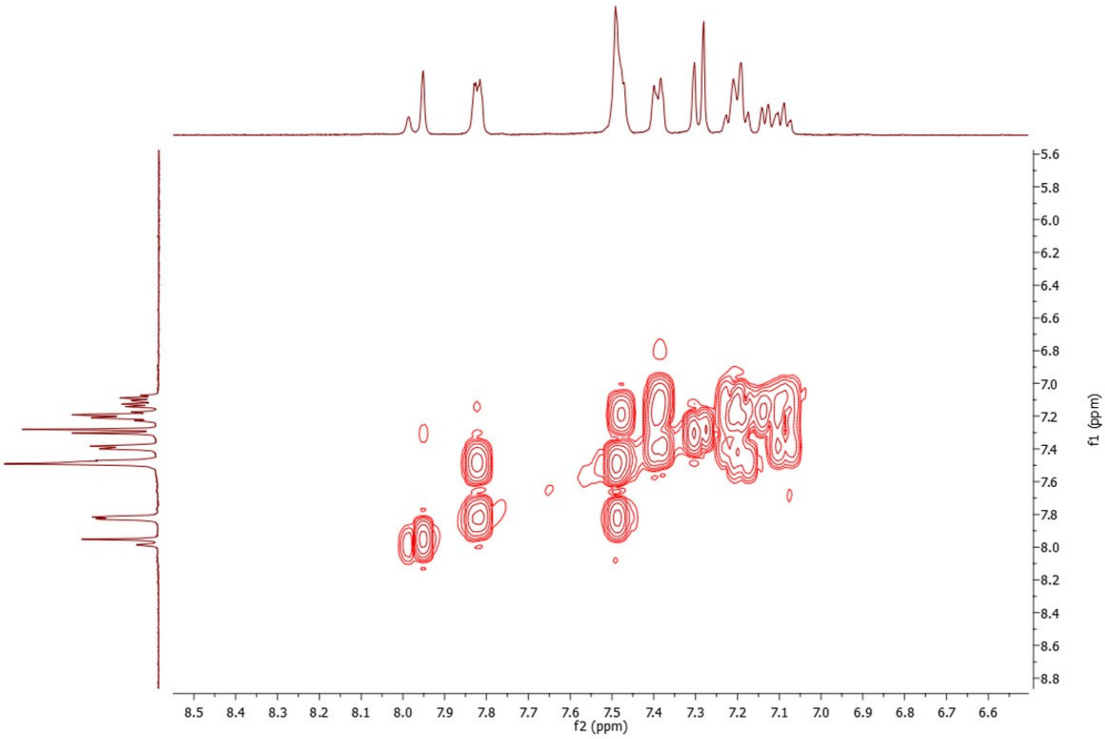

Figure S4. $^1H,^1H$ COSY spectrum of compound **1** ($CDCl_3$).

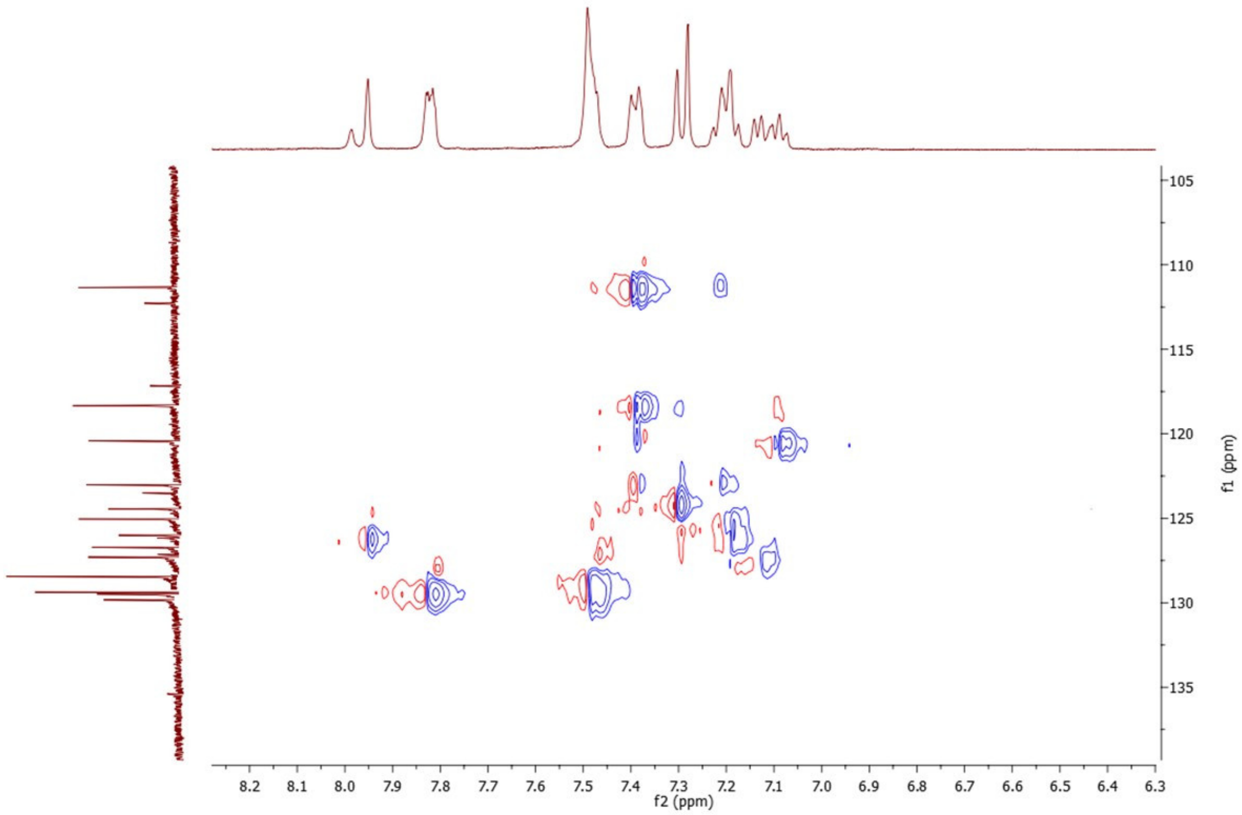

Figure S5. $^{1}H,^{13}C$ HSQC spectrum of compound **1** ($CDCl_3$).

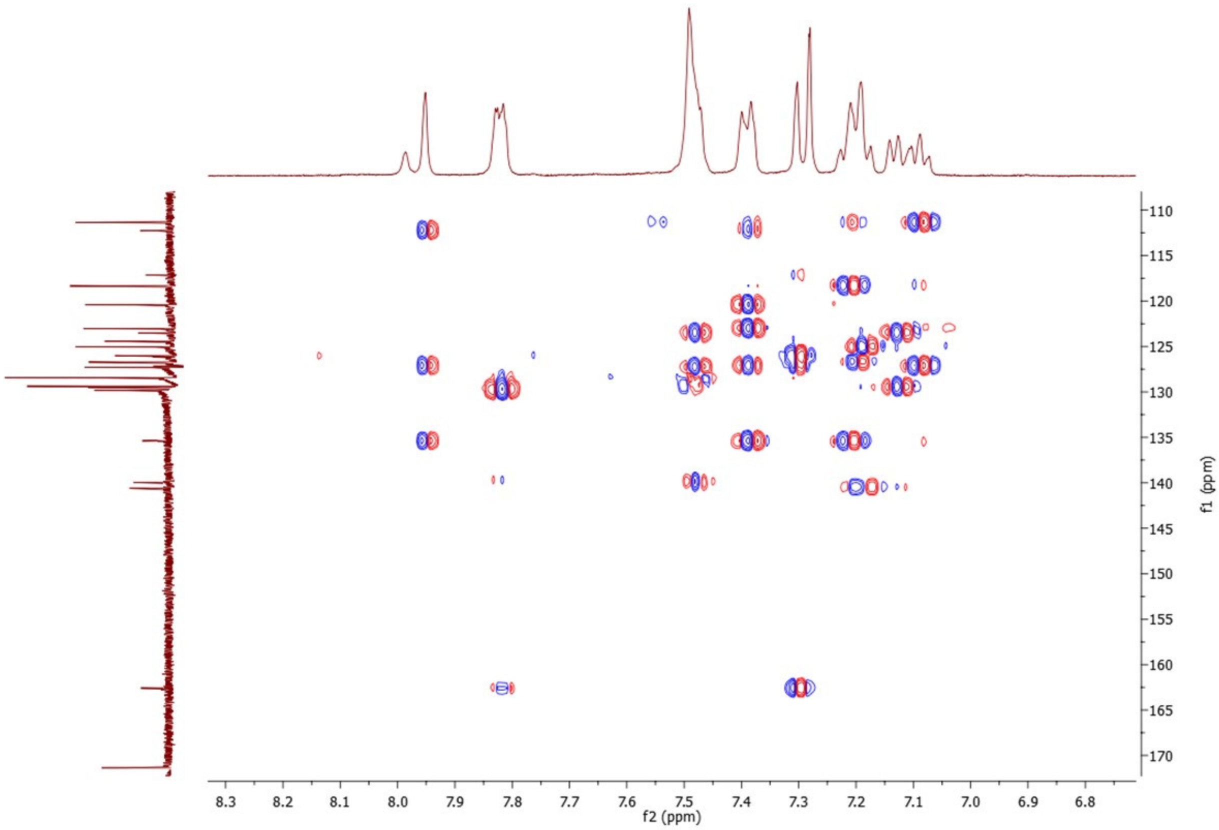

Figure S6. $^{1}H,^{13}C$ HMBC spectrum of compound **1** ($CDCl_3$).

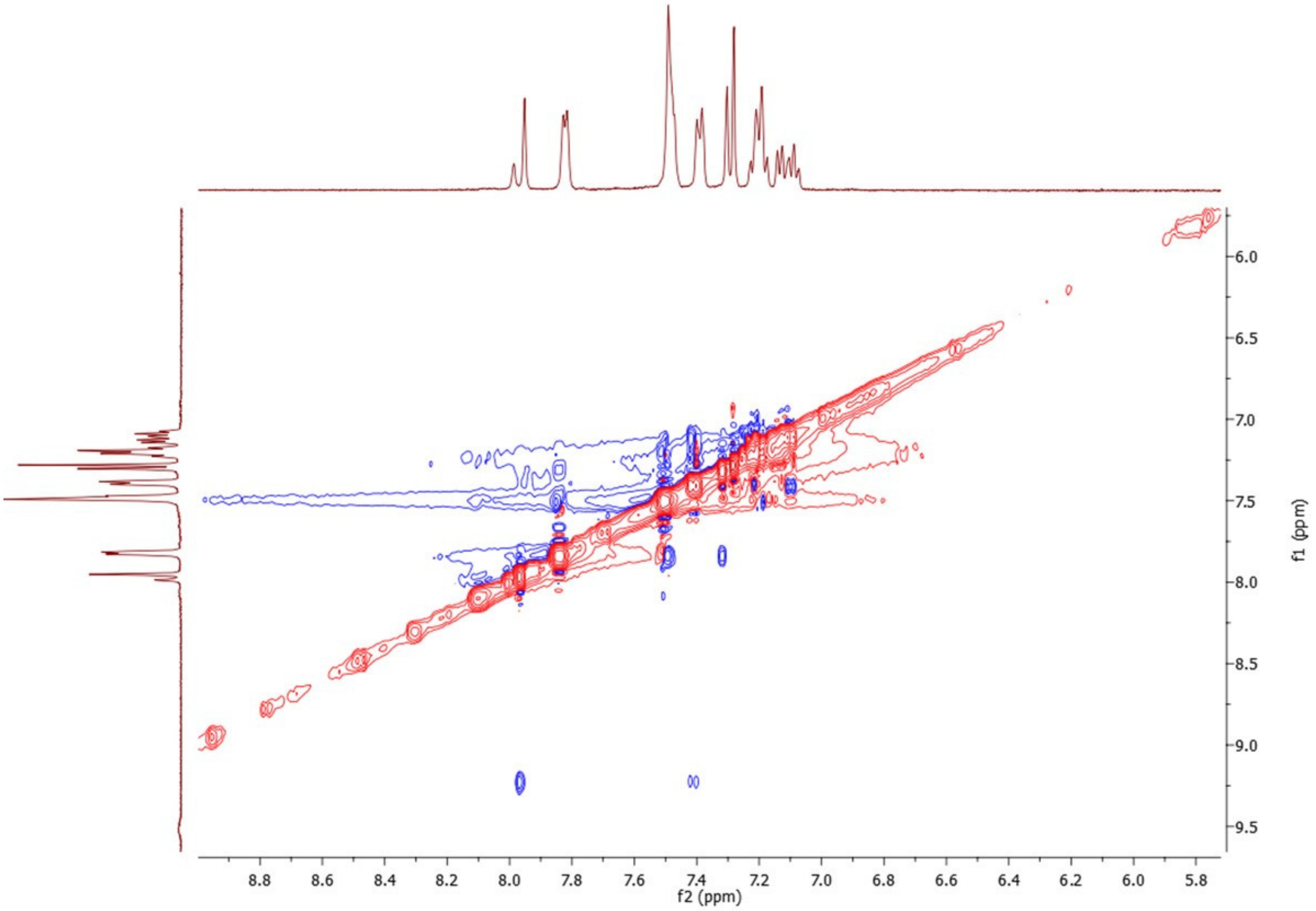

Figure S7. NOESY spectrum of compound **1** ($CDCl_3$).

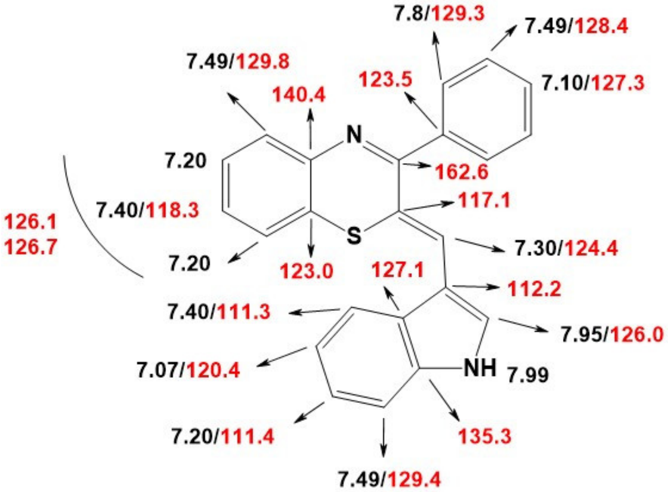

Figure S8. ^{1}H (black) and ^{13}C (red) NMR resonances of cyanine **1**.

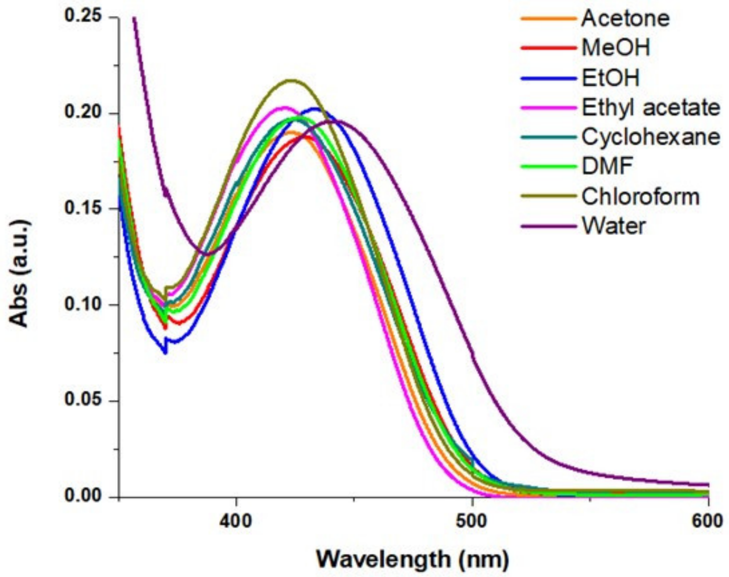

Figure S9. UV-vis absorption spectra of cyanine **1** (100 μM) in different organic solvents.

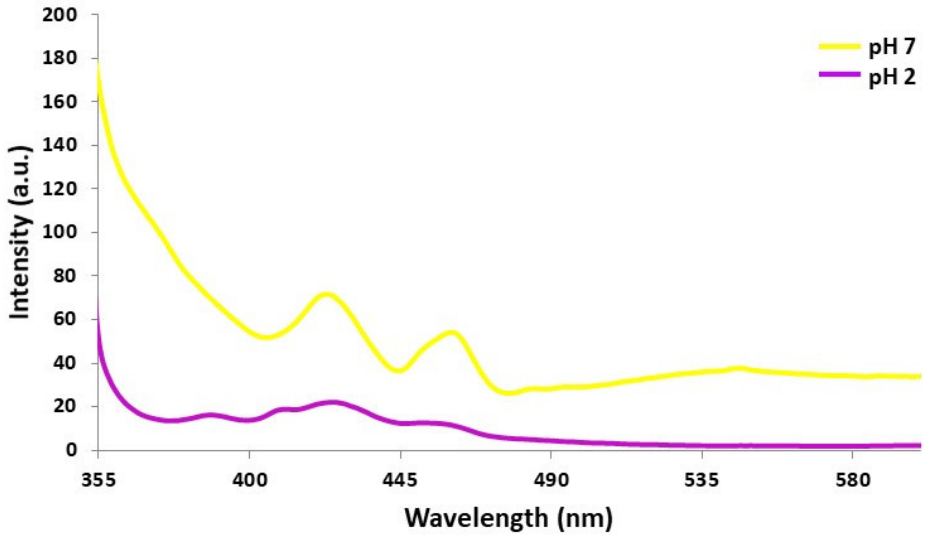

Figure S10. Emission spectra of the neutral and protonated forms of cyanine **1** in aqueous buffers at different pHs.

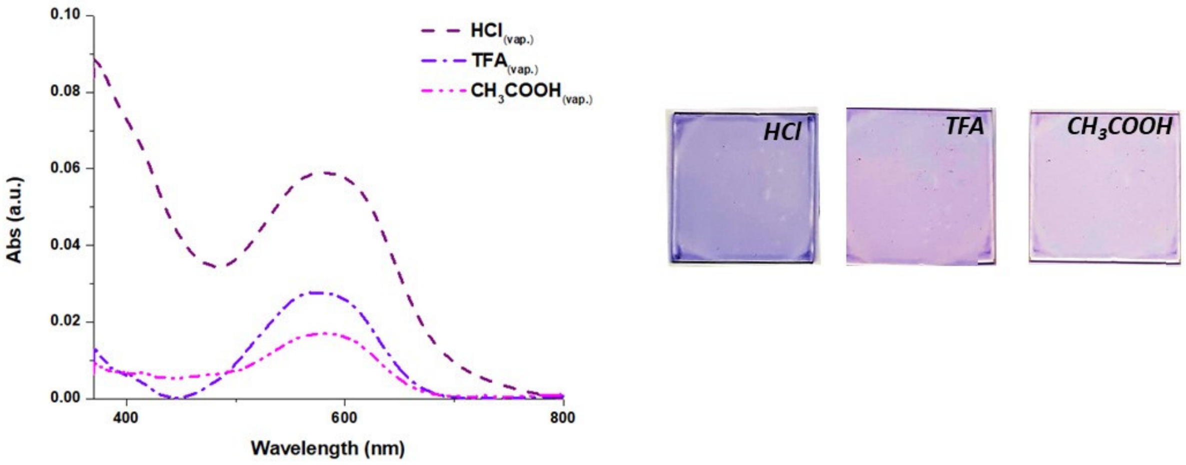

Figure S11. UV-vis spectra and digital pictures of glass slides, coated with 1, after exposure to acidic vapours.
